# Mie-optimized PMMA particles for fully polymer-based radiative cooling coatings with high reflectance and hydrophobicity†

**DOI:** 10.1039/d5ra01834j

**Published:** 2025-06-05

**Authors:** Chong-En Hu, To-Yu Wang, Duo-Syuan Lin, Ming-Wei Chen, Chao-Wei Huang

**Affiliations:** a Department of Engineering Science, National Cheng Kung University Tainan 70101 Taiwan huangcw@gs.ncku.edu.tw n96111435@gs.ncku.edu.tw n98121070@gs.ncku.edu.tw n98121088@gs.ncku.edu.tw n96134530@gs.ncku.edu.tw

## Abstract

Passive radiative cooling materials achieve cooling without energy consumption by reflecting sunlight and emitting thermal radiation to the cold outer space (3 K). This study synthesizes poly(methyl methacrylate) (PMMA) particles with Mie scattering properties through emulsifier-free emulsion and dispersion polymerization to control particle size, enhancing their scattering and reflectivity. A high-emissivity polydimethylsiloxane (PDMS) matrix is used to fabricate a composite material, enabling complementary radiative effects between PMMA and PDMS within the atmospheric transparency window, thus enhancing the cooling efficiency. The impact of PMMA particle size on reflectance was investigated, and the performance of varying PMMA–PDMS ratios in radiative cooling was assessed. Experimental results indicate that PMMA particles synthesized in-house, particularly PMMA-1, exhibit optimal radiative properties, with a reflectance of 93.7% and emissivity of 93.2%. The composite A_0.7_S_0.3_ coating with an optimized PMMA : PDMS ratio of 7 : 3 exhibited a solar reflectance of 96.9% and an emissivity of 94.0% within the atmospheric window wavelength range. Outdoor testing showed that this A_0.7_S_0.3_ coating achieves an average temperature reduction of 3.4 °C and a maximum of 8.6 °C below ambient temperature, outperforming commercial coatings. Additionally, water contact angle measurements indicated hydrophobicity (111.4°), suggesting self-cleaning potential. These findings reveal that the A_0.7_S_0.3_ coating exhibits promising cooling performance, self-cleaning capabilities, and cost-effectiveness, highlighting its potential for practical applications in radiative cooling.

## Abstract 2

PMMA–PDMS (A_0.7_S_0.3_) coating has 96.9% reflectance and 94.0% emissivity, and has a 111.4° water contact angle, showing hydrophobicity. Most importantly, PMMA–PDMS (A_0.7_S_0.3_) coating lowers the temperature by 8.60 °C below ambient.

## Graphical abstract

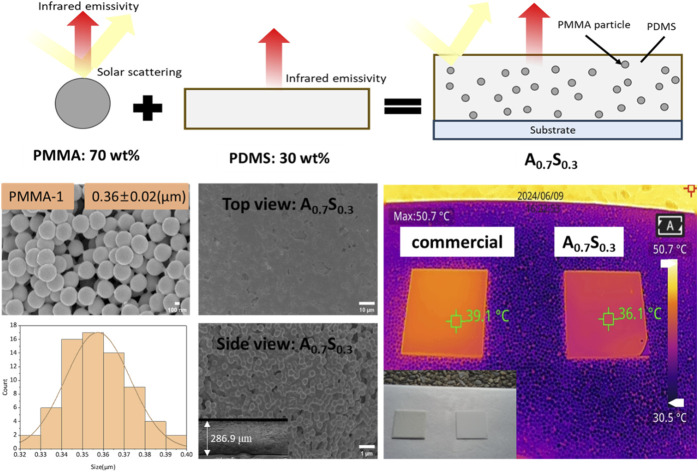

## 1. Introduction

The rapid development of technology and industry has increased energy consumption and greenhouse gas emissions, exacerbating global warming and urban heat island effects.^1^ This has intensified the reliance on air conditioning, further aggravating the greenhouse effect. Passive radiative cooling has emerged as an energy-free technology by reflecting sunlight (0.3–2.5 μm) and emitting thermal radiation through the atmospheric window (8–13 μm).^2^ Passive radiative cooling has found practical applications in textiles,^3^ architectural coatings,^4^ vehicles,^5^ and cooling films,^6^ establishing itself as a novel solution for mitigating greenhouse and urban heat island effects.

Raman *et al.* proposed a multilayer photonic structure for passive radiative cooling,^7^ which has since inspired diverse designs, including multilayers,^8,9^ metamaterials,^10–12^ porous coatings,^13–15^ metal-backed composites,^16–18^ and randomly dispersed particle structures.^19–23^ For instance, Zou *et al.* fabricated square-patterned gratings on chromium- and nickel-coated silicon wafers, achieving a cooling effect of 10 °C below ambient temperature.^11^ However, these complex, costly, and metal-dependent structures limit large-scale applications. Therefore, practical radiative cooling demands low-cost, high-performance materials that are compatible and easy to fabricate.

The coating-based radiative cooling is the most practical and cost-effective approach for large-scale applications. These coatings typically combine high-reflectivity inorganic particles, such as Al_2_O_3_,^24^ SiO_2_,^25^ TiO_2_,^26^ BaSO_4_,^27^ and CaCO_3_,^28^ with polymeric binders of high infrared emissivity, including PDMS,^29^ PMMA,^30^ and PVDF.^31^ For example, Chae *et al.* developed an acrylic-based coating with Al_2_O_3_ and SiO_2_, achieving 94.1% solar reflectance and 93.5% emissivity, resulting in a 5.1 °C cooling effect under sunlight.^4^ Liu *et al.* fabricated a sustainable BaSO_4_-ethyl cellulose coating using green solvents, enabling easy application and material recyclability.^32^

Compared to traditional inorganic additives, polymer additives offer advantages such as lower solar absorption, cost-effectiveness, lightweight properties, and abundant absorption peaks in the atmospheric window.^33^ Jiang *et al.* recently developed fully polymer-based radiative cooling paints using PTFE combined with PDMS, which provide solar reflectance and emissivity as high as 92.0% and 93.0%, respectively.^34^ Furthermore, Jiang *et al.* developed another all-polymer coating by combining PVDF with PDMS, achieving an average reflectance of 90.6% and an average emissivity of 98.1%.^35^ This demonstrates that enhancing the reflectance of all-polymer coatings is challenging; however, reflectance performance is crucial for the effectiveness of radiative coolers. The cooling material should absorb less than 10% of incident solar radiation and minimize UV absorption to prevent yellowing and performance degradation over time.^36^ Due to its favorable refractive index and tunable particle morphology,^37^ PMMA has been widely applied in optical^38^ and radiative cooling applications.^39^ PMMA is well-suited for enhancing solar reflectance through Mie scattering. The optical properties and cooling performances of different types of radiative cooling composite coatings in the literature were summarized in Table S1.† Unlike previous studies, the present work utilizes PMMA particles as the primary scattering agents to enhance solar reflectance. By controlling the particle size, effective Mie scattering can be induced within the solar spectrum,^25^ thereby reducing overall solar absorption and improving the radiative cooling performance.

In this study, we present a fully polymer-based PMMA–PDMS radiative cooling coating designed to maximize solar reflectance and UV reflectivity. PMMA particles, known for their processability and excellent thermal emissivity,^40^ are employed as the primary scattering agents. By tailoring the particle size, strong Mie scattering within the solar wavelength range is induced, thereby achieving enhanced solar reflectance.^41^ These spherical PMMA particles are synthesized with optimized dimensions to ensure maximum scattering efficiency. PDMS serves as the binder due to its film-forming capability^42^ and its spectral complementarity with PMMA in the atmospheric window,^43^ resulting in a composite coating with both high solar reflectance and infrared emissivity. Furthermore, outdoor cooling performance tests and water contact angle measurements confirm that the PMMA–PDMS coating offers a cost-effective, high-performance, and substrate-compatible solution, demonstrating strong potential for energy-efficient cooling applications.

## 2. Experimental

### 2.1 Materials

The materials used in this study were high-purity reagents, each sourced from reputable suppliers. Methyl methacrylate (MMA, C_5_H_8_O_2_, CAS: 80-62-6) with a purity of 99.9% and methanol (CH_3_OH, CAS: 67-56-1) with a 99.9% purity were purchased from AENCORE. Potassium persulfate (KPS, K_2_S_2_O_8_, CAS: 7727-21-1, 98.0% purity) was obtained from SHOWA. Polydimethylsiloxane (PDMS, (C_2_H_6_OSi)_*n*_) was provided by Dow Chemical and used without further purification. Polyvinylpyrrolidone (PVP, (C_6_H_9_NO)_*n*_, CAS: 9003-39-8) with a ≥99% purity was sourced from ALDRICH. Azobisisobutyronitrile (AIBN, C_8_H_12_N_4_, CAS: 78-67-1, 99.7% purity) was purchased from Otsuka Chemical, and *tert*-butanol (*t*-BuOH, C_4_H_10_O, CAS: 75-65-0) with a purity of 99.9% was acquired from Scharlau. Finally, 3-aminopropyltriethoxysilane (APTES, C_9_H_23_NO_3_Si, CAS: 919-30-2) with a 99.8% purity was provided by Thermo Scientific. Each reagent was used as received without any additional purification.

### 2.2 Synthesis of PMMA particles

PMMA particles of varying sizes were synthesized using two polymerization methods: emulsifier-free emulsion polymerization and dispersion polymerization.^44^ For the emulsifier-free emulsion polymerization, the monomer MMA and deionized (DI) water were stirred and preheated for 30 min to ensure the system reached the reaction temperature of 80 °C. The KPS initiator was then added, and the mixture was stirred at 300 rpm for 2 h at 80 °C, after which the reaction was complete.^45^ The reaction solution was subsequently dried in a vacuum oven to obtain PMMA-1 particle powder. The second method, dispersion polymerization, involved initially adding methanol, DI water, and the PVP dispersant to the reactor, followed by stirring and preheating for 30 min to ensure the system reached 60 °C. The monomer MMA and the initiator AIBN were then introduced, and the reaction proceeded at 300 rpm for 6 h at 60 °C. After completion, the reaction mixture was centrifuged and washed five times, then dried in a vacuum oven to yield PMMA-2 and PMMA-3 particle powders. The PMMA-2 and PMMA-3 differ in size due to variations in the methanol-to-water ratio of 2 : 1 and 7 : 3, respectively. The actual sample images of PMMA-1, PMMA-2, and PMMA-3 are provided in Fig. S1.†

### 2.3 Synthesis of PMMA–PDMS (A_*X*_S_*Y*_) coatings

Radiative cooling coatings were prepared by blending PMMA particles with PDMS to form a fully polymer-based PMMA–PDMS composite coating. The coating slurry was applied onto glass substrates using a blade-coating technique and was then allowed to dry at room temperature to form the radiative cooling layer. Initially, the desired amount of PMMA powder, the solvent *tert*-butanol, and the APTES dispersing agent were mixed and stirred at 500 rpm for 6 h. Separately, a second mixture containing PDMS, *tert*-butanol, and APTES was prepared and stirred at 500 rpm for 2 h. These two solutions were then combined and stirred together at 500 rpm for an additional 2 h to ensure thorough mixing. Subsequently, a curing agent was added, and the mixture was stirred at 500 rpm for 1 h to initiate cross-linking in the PDMS matrix. The completed radiative cooling slurry was then drop-cast onto glass substrates and further spread evenly using a blade-coating device. To investigate the effects of varying PMMA and PDMS content, coatings were prepared with different weight ratios of PMMA to PDMS. The resulting samples were named following the format A_*X*_S_*Y*_, where “A” and “S” represent the tail letters of PMMA and PDMS, respectively. “_*X*_” indicates the weight fraction of PMMA particles, and “_*Y*_” represents the weight fraction of PDMS, where *Y* = 1 − *X* and *X* = 0.5, 0.6, 0.7, 0.8. The actual coating images provided are shown in Fig. S2.† To further evaluate the cost-effectiveness and scalability of our PMMA–PDMS coatings, a comparison with commercial white coating (CWC, Nippon Pylox, #102 color) is summarized in Table S2.†

### 2.4 Outdoor experimental setup

**Fig. 1:**
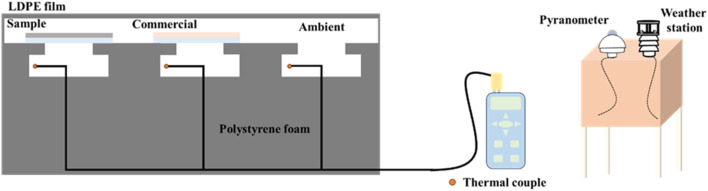
Radiative cooling device design.

The outdoor experimental setup utilized a polystyrene foam body with high thermal insulation properties to minimize environmental heat conduction effects on the cooling device. To evaluate the cooling effect of the radiative cooling coating developed in this study, several chambers were incorporated within the foam body. Each chamber was equipped with a K-type thermocouple (TM-947SD, Taiwan) connected to a temperature recorder, set to log temperature changes every 30 s. This setup was designed to simulate indoor environmental temperature variations by monitoring the temperature within the chambers. For testing, the coating was applied over the top of the chambers, with one chamber left uncoated to serve as a control representing ambient temperature conditions. To further prevent heat convection interference with the cooler, the top of the setup was covered with low-density polyethylene (LDPE). Due to the numerous external factors in an outdoor environment (such as humidity, wind, ambient temperature, and solar irradiance) that could impact measurement accuracy, a CS-WS61 weather station (Taiwan) and a PYR2-420 pyranometer (Italy) were employed. A schematic illustration of the setup is provided in Fig. 1, with actual setup images shown in Fig. S3.†

### 2.5 Calculations of net cooling performance

The cooling performance of the radiative cooler involves an energy balance comprising four main power components: the radiative power emitted by the cooler (*P*_rad_), the absorbed solar irradiance (*P*_sun_), the atmospheric thermal radiation absorbed (*P*_atm_), and the energy losses due to non-radiative heat transfer processes, including convection and conduction (*P*_cond+conv_). The net radiative cooling power (*P*_net_) is obtained by balancing these energy contributions. The theoretical value of *P*_net_ can be calculated using the equation proposed by Raman *et al.* in 2014, which defines the balance between these thermal processes to determine the net cooling effect achievable under specific environmental conditions:^7^

1 *P*_net_(*T*) = *P*_rad_(*T*) − *P*_sun_ − *P*_atm_(*T*_atm_) − *P*_cond+conv_

In the net radiative cooling power equation, each term represents a specific physical contribution and can be calculated based on previous studies.^25^ According to eqn (1), achieving effective cooling requires that *P*_net_(*T*) > 0; only then can the cooler provide a net cooling effect. This necessitates maximizing the radiative cooling power while minimizing the absorption of solar irradiance and atmospheric radiation. Additionally, non-radiative heat losses, such as convection and conduction, should be kept to a minimum. Together, these conditions ensure optimal cooling performance. Conversely, if the net radiative cooling power is less than zero, it indicates that the cooler is unable to deliver an effective cooling effect.

### 2.6 Characterization

The morphology of PMMA particles and the PMMA–PDMS(A_0.7_S_0.3_) coatings was observed by scanning electron microscopy (SEM, ZEISS AURIGA) at 10 kV. An energy-dispersive X-ray spectroscopy (EDS) attached to the SEM was used to assess the elemental distribution in the A_0.5_S_0.5_–A_0.8_S_0.2_ coatings. Fourier-transform infrared (FT-IR) transmittance spectra of the PMMA particles and the A_0.5_S_0.5_ to A_0.8_S_0.2_ coatings were obtained over a wavenumber range from 4000 to 600 cm^−1^ using a Nicolet 6700 FT-IR spectrometer (Thermo Scientific) in attenuated total reflectance (ATR) mode. The water contact angle (WCA) of the PDMS coating, a commercial coating, and the A_0.5_S_0.5_ to A_0.8_S_0.2_ coatings was measured with an optical contact angle instrument (Dataphysics/OCA15EC) using a 5 μL droplet of DI water. Surface temperatures of the A_0.7_S_0.3_ coatings and commercial coatings were monitored with an M-series handheld thermal camera (IRay Technology Co., Ltd/M305). Reflectance measurements for the PMMA particles and the A_0.5_S_0.5_ to A_0.8_S_0.2_ coatings across 0.3–2.5 μm (the solar spectrum range) were conducted using Jasco V-670 ultraviolet-visible-near-infrared (UV-Vis-NIR) spectroscopy equipped with a Jasco ISN723 diffuse integrating sphere. The average solar reflectance was determined as follows:^46^2
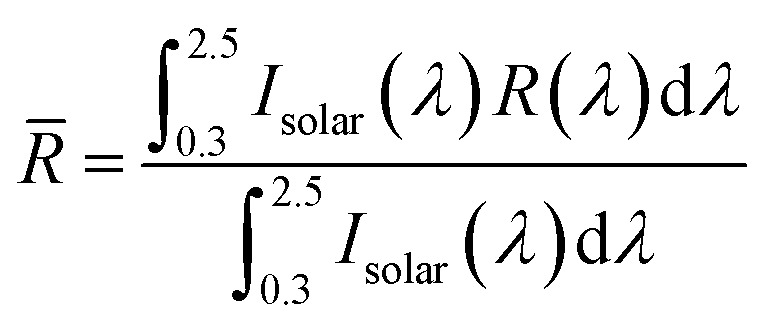
where *R̄* is the average reflectivity in the range from 0.3–2.0 μm, *I*_solar_ is the global solar intensity spectrum provided by ASTM G173.^47^

The emissivity of the PMMA particles and the A_0.5_S_0.5_ to A_0.8_S_0.2_ coatings within 4–20 μm (the mid and long-wavelength range) was measured using the Nicolet 5700 FT-IR spectrometer (USA) with a PIKE-048-3350 integrating sphere (USA). All spectral emissivity was defined through Kirchhoff's law of radiation.^48^

3 *αλ* = *ελ*

where *α* is the absorptivity at wavelength *λ*, and *ε* is the emissivity at *λ*.

The average emissivity within 8–13 μm (the atmosphere window range) can be calculated as:^49^4
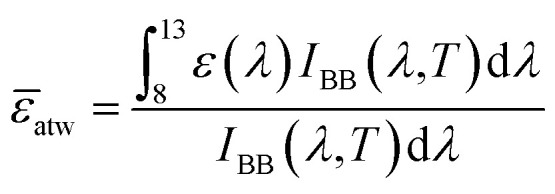
where *ε̄*_atw_ is the average emissivity in the atmosphere window range, and *I*_BB_ is the spectral intensity by the blackbody.

## 3. Results and discussions

### 3.1 Characterization of the PMMA-1 to PMMA-3 particles

**Fig. 2:**
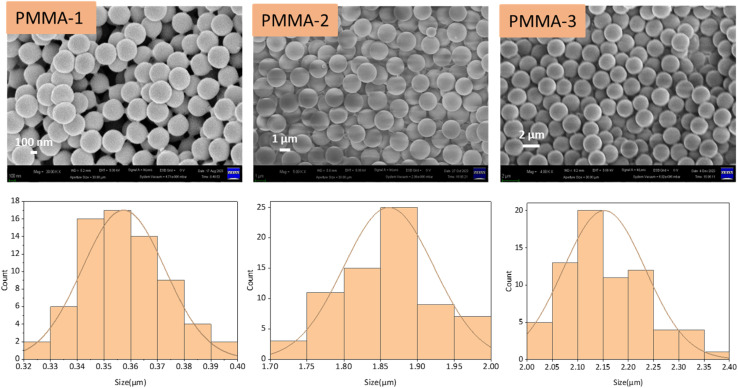
SEM images and particle size distribution of PMMA-1, PMMA-2, and PMMA-3 particles.

In this study, PMMA particles of varying sizes were synthesized using a straightforward emulsifier-free emulsion polymerization (PMMA-1) and a dispersion polymerization method (PMMA-2 and PMMA-3). As shown in Fig. 2, their SEM images reveal the size and surface morphology of the PMMA microspheres, and the particle size distributions are obtained by using ImageJ. SEM observations indicate that PMMA particles synthesized by both methods exhibit spherical shapes and smooth surfaces. PMMA-1 particles produced by the emulsifier-free emulsion polymerization had a smaller average diameter of 0.36 ± 0.02 μm, whereas PMMA-2 and PMMA-3 synthesized *via* dispersion polymerization were larger, with diameters of 1.86 ± 0.06 μm and 2.15 ± 0.08 μm, respectively. The larger particle sizes obtained through dispersion polymerization are attributed to the higher affinity between the monomer and methanol, which supports more extensive chain growth, thereby yielding larger particle sizes.^50^ This trend aligns with an increase in particle size as the methanol content is raised. Notably, the particle sizes achieved in this study fall within the solar wavelength range, suggesting that each synthesized PMMA particle has the potential to exhibit Mie scattering characteristics effectively.

**Fig. 3:**
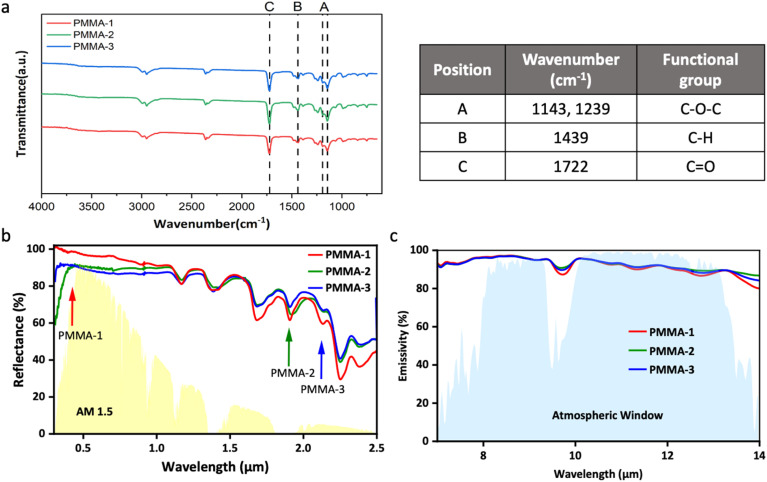
(a) FTIR spectra and parameters, (b) solar reflection spectrum, and (c) infrared emission spectrum of PMMA-1, PMMA-2, and PMMA-3 particles.

The molecular structure and functional groups of the PMMA samples were characterized by FT-IR spectroscopy. The spectral range from 1250 to 770 cm^−1^, known as the atmospheric window, is particularly important. According to Kirchhoff's law, materials with strong absorption characteristics in this range also demonstrate strong emissive properties. In the FT-IR spectra, prominent absorption peaks for PMMA were observed at 1143 cm^−1^ and 1239 cm^−1^ (labeled as peaks A), along with a weaker peak at 1439 cm^−1^ (peak B), and a strong peak at 1722 cm^−1^ (peak C). Notably, the peaks at 1143 cm^−1^ and 1239 cm^−1^ fall within the atmospheric window, indicating that PMMA has substantial absorption and emissive characteristics in the desired wavelength range. These peaks correspond to C–O–C stretching vibrations, aligning with previous literature data on PMMA,^51^ thus confirming the successful synthesis of PMMA particles in this study, as illustrated in Fig. 3(a).

When the size of particles or pores is comparable to the wavelength of incident light, strong Mie scattering is induced.^52,53^ Therefore, by controlling the particle size, the scattering range can be effectively tuned to achieve optimal solar reflectance.^54^ As shown in Fig. 3(b), the PMMA-1 particles, with an average size of 0.36 μm, satisfied the condition for effective Mie scattering within the solar spectrum, resulting in superior reflectance in the 0.3–1.2 μm wavelength range. In contrast, PMMA-2 and PMMA-3, with particle sizes of 1.86 μm and 2.15 μm, respectively, exhibited enhanced reflectance in the 1.7–2.5 μm region, consistent with the Mie scattering behavior of larger particles. However, PMMA inherently exhibits UV absorption,^55^ which limits the UV reflectance of larger PMMA particles. To address this, the particle size of PMMA-1 was controlled within 300–400 nm to leverage Mie scattering for enhancing UV reflectance, thereby mitigating UV absorption and improving overall solar reflectance performance.

The emission spectra of PMMA particles, shown in Fig. 3(c), demonstrate the emissive performance of PMMA-1, PMMA-2, and PMMA-3. Although different polymerization methods were used, each PMMA particle size displayed high emissive performance (>93%) within the atmospheric window due to the rich vibrational peak features of PMMA in this range. Based on the combined reflectance and emissivity results, PMMA-1 exhibited superior radiative cooling performance, making it the preferred additive for subsequent coating formulations. Detailed results for the reflectance and emissivity of each PMMA particle are summarized in Table S3.†

### 3.2 Characterization of the PMMA–PDMS coatings

**Fig. 4:**
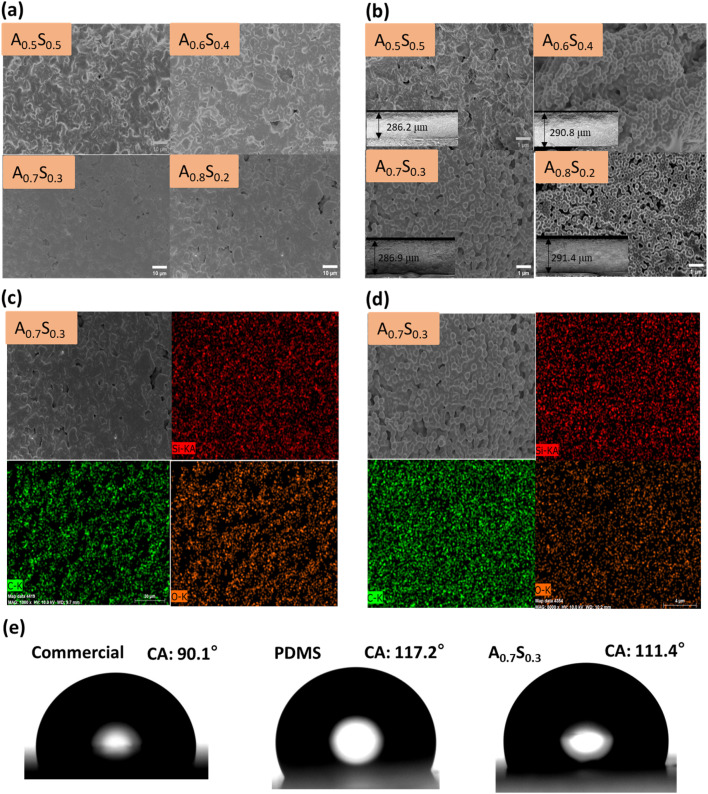
(a) Top-sectional and (b) cross-sectional SEM images of coatings with different ratios (A_0.5_S_0.5_, A_0.6_S_0.4_, A_0.7_S_0.3_, and A_0.8_S_0.2_); (c) top-sectional and (d) cross-sectional EDS-mapping of A_0.7_S_0.3_; (e) water contact angles of PMMA, PDMS, commercial, and A_0.7_S_0.3_ coatings.

For effective daytime radiative cooling, coatings must exhibit both high solar reflectance and strong infrared emissivity. To achieve this, PMMA and PDMS were combined in varying ratios from 0.5 : 0.5 to 0.8 : 0.2. As shown in Fig. S2,† the A_0.5_S_0.5_ to A_0.8_S_0.2_ coatings maintained good film integrity without noticeable cracking or peeling, indicating excellent film-forming capability.^56^ This is primarily attributed to the presence of PDMS, which serves as a binder with superior film-forming properties, effectively embedding and securing PMMA particles within the matrix. To further examine the coating uniformity, SEM was employed to observe the surface morphology and cross-sectional structure. As shown in Fig. 4(a), the top-sectional SEM images revealed that decreasing PDMS content led to an increase in surface porosity. This phenomenon is attributed to the reduction in binding strength provided by PDMS, resulting in weaker cohesion between PMMA particles and the formation of voids. These findings confirm the critical role of PDMS in promoting uniform particle distribution and mechanical integrity within the coating. Cross-sectional SEM views further demonstrated that higher PDMS content in the A_0.5_S_0.5_ masked the PMMA particles. As the PDMS content decreased, the PMMA particles became more exposed, as seen in the A_0.6_S_0.4_, although minor particle aggregation was noted. At the A_0.7_S_0.3_ ratio, the PMMA particles were well-dispersed with minimal aggregation. In contrast, further reduction of the A_0.8_S_0.2_ ratio led to visible porosity due to insufficient binder content, as shown in Fig. 4(b).

To confirm the dispersion and composite structure of the coatings, EDS mapping was employed. The EDS mapping images of the A_0.7_S_0.3_ coating, both top-view and cross-sectional (Fig. 4(c) and (d), with additional ratios shown in Fig. S4 and S5†), revealed the presence of Si, C, and O elements. The Si signals correspond to PDMS's Si–O and Si–CH_3_ bonds, while the C signals are associated with PMMA's C=O, C–H, and C–O–C bonds. The EDS results indicated good dispersion without significant aggregation, with clear signals of both PMMA and PDMS in the top and cross-sectional views, confirming successful integration.

Given that radiative coolers are typically used outdoors, resistance to contamination and maintenance of cooling performance is essential. Therefore, hydrophobicity, which aids in self-cleaning, is a crucial indicator. The WCA was measured to assess the coating's hydrophobic properties, with results shown in Fig. 4(e). Direct WCA measurements of PMMA powder were challenging; hence, values from PMMA thin films in the literature were referenced.^57^ PMMA films displayed a WCA of 67.8°, indicating hydrophilicity, whereas PDMS exhibited a WCA of 117.2°, characterizing it as hydrophobic. The blended A_0.7_S_0.3_ coating demonstrated a WCA of 111.4°, falling into the hydrophobic range. This WCA surpassed that of commercial white paint, which showed a WCA of 90.1°. The transformation of hydrophilic PMMA into a hydrophobic coating is attributed to PDMS's low surface energy due to C–H bonds, imparting the coating with hydrophobic properties.

**Fig. 5:**
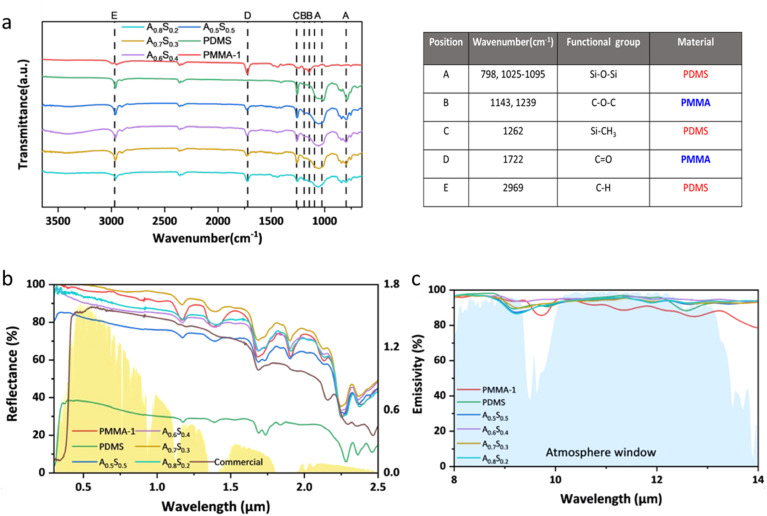
(a) FTIR spectra and parameters, (b) solar reflection spectrum, and (c) infrared emission spectrum of the PMMA, PDMS, commercial, A_0.5_S_0.5_, A_0.6_S_0.4_, A_0.7_S_0.3_, and A_0.8_S_0.2_ coatings.

The molecular structure and functional groups of the various PMMA–PDMS coating formulations were characterized using FT-IR spectroscopy. The spectral range of 1250–770 cm^−1^ corresponds to the atmospheric window (8–13 μm), a key region for evaluating radiative cooling properties. FT-IR spectra showed distinct absorption peaks within this range at 798 cm^−1^ (A),^58^ 1025–1095 cm^−1^ (A),^58,59^ 1143 cm^−1^ (B),^51^ and 1239 cm^−1^ (B).^51^ The peaks at 798 cm^−1^ and 1025–1095 cm^−1^ are attributed to the Si–O–Si bonds in PDMS, while the peaks at 1143 cm^−1^ and 1239 cm^−1^ are due to the C–O–C bonds in PMMA. According to Kirchhoff's law, these absorption characteristics confirm the coating's strong emissive properties within the atmospheric window. Additional absorption peaks at 1262 cm^−1^ (C),^60^ 1722 cm^−1^ (D),^51^ and 2969 cm^−1^ (E)^61^ were also observed, corresponding to the Si–CH_3_ and C–H bonds in PDMS, and the C=O bond in PMMA, confirming successful integration of PMMA particles and PDMS within the composite, as shown in Fig. 5(a).

To ensure consistency in the optical property measurements, the thickness of all coatings was controlled within the range of 285–291 μm (Table S4†). This precaution was taken to prevent any influence of excessive or insufficient thickness on the accuracy of reflectance and emissivity measurements.^54^ To evaluate the optical performance of the PMMA–PDMS coatings, a commercial white coating (CWC, Nippon Pylox, #102 color) was employed as a reference. This commercial coating primarily consists of titanium dioxide (TiO_2_) pigments and an acrylic resin binder. Reflectance measurements revealed that commercial coatings exhibit substantial UV solar absorption due to the use of TiO_2_ particles, which absorb solar radiation in the UV range. Consequently, the commercial coating's average solar reflectance was limited to 79.7%. Pure PDMS, lacking reflective fillers or pores, exhibited an even lower average solar reflectance of 34.3%. In contrast, the custom coatings (A_0.5_S_0.5_ to A_0.8_S_0.2_) showed a trend of increasing solar reflectance with higher PMMA content. A_0.7_S_0.3_ achieved the highest average solar reflectance of 96.9%, though further increasing PMMA content to A_0.8_S_0.2_ led to a decrease, likely due to irregular pore formation reducing overall reflectance, as shown in Fig. 5(b).

The radiative spectra in the atmospheric window range demonstrated that both PMMA and PDMS exhibit strong thermal emissivity. Consequently, the custom coatings (A_0.5_S_0.5_ to A_0.8_S_0.2_) achieved emissivity levels above 93.5%, confirming their suitability for radiative cooling. Based on these results, the A_0.7_S_0.3_ coating, which combines high solar reflectance with excellent emissivity, was selected for further outdoor testing, as illustrated in Fig. 5(c). Detailed reflectance and emissivity measurements for each coating ratio are also provided in Table S4.†

In this study, we aimed to develop a radiative cooling coating with high compatibility and broad applicability across various surfaces. Therefore, the material compatibility of the coating with different substrates is critical.^62^ To validate the versatility and scalability of the PMMA–PDMS coatings, the prepared coatings were applied onto various substrate materials, including glass, aluminum, copper, and wood, as illustrated in Fig. S6.† The experimental results confirm that the PMMA–PDMS coatings adhere effectively to all tested surfaces, demonstrating their excellent material compatibility and wide applicability. These findings indicate the coating's strong potential for practical deployment on diverse building surfaces for radiative cooling purposes.

### 3.3 Outdoor radiative cooling performance of A_0.7_S_0.3_ coatings

**Fig. 6:**
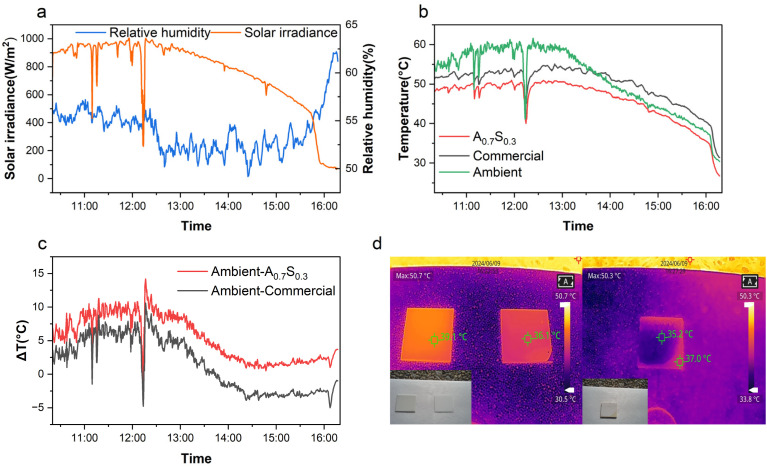
Outdoor radiative cooling performance test on May 10, 2024, between 10 : 10 and 15 : 10 in Tainan, Taiwan. (a) Meteorological parameters: relative humidity and solar irradiance; (b) outdoor radiative cooling experiment with ambient, A_0.7_S_0.3_, and commercial coatings; (c) temperature difference plot between ambient and A_0.7_S_0.3_ and commercial coatings; (d) left: infrared thermal images of A_0.7_S_0.3_ and commercial coatings applied on glass; right: infrared thermal images of A_0.7_S_0.3_ coatings applied on woods.

Outdoor experiments were conducted using a custom-built experimental setup to confirm the outdoor cooling performance of the A_0.7_S_0.3_ coatings. As shown in Fig. 6(a), the outdoor environmental factors, including relative humidity and solar irradiance, were recorded. It presents the climate conditions measured on May 10, 2024, from 10 : 10 to 15 : 10, with an average humidity of 54.4% and an average wind speed of 1.57 m s^−1^. Fig. 6(b) illustrates the temperature variations of the PMMA–PDMS coatings, commercial white coating, and the ambient temperature within the setup, with the day's solar irradiance also plotted for reference. A slight greenhouse effect was observed due to the LDPE film isolating the experimental chamber from external convective heat, resulting in marginally elevated measured temperatures.^63^ It indicates that, under high solar irradiance (*I*_solar_) of up to 1005 W m^−2^, the cooling performance of the custom PDRC coating consistently outperformed both the ambient temperature and the commercial coating.

To highlight the cooling efficacy, a temperature differential plot (Fig. 6(c)) was shown, comparing the ambient temperature with the A_0.7_S_0.3_ coating and commercial white coating. The results show that the A_0.7_S_0.3_ coating provided a cooling effect of 1.92 °C better than the commercial coating and of 3.39 °C below the ambient temperature, with a maximum temperature difference of up to 8.60 °C compared to the ambient. Additionally, infrared thermal imaging was employed to monitor the real-time surface temperature of substrates coated with the A_0.7_S_0.3_ coating, as shown in Fig. 6(d). On the left of Fig. 6(d), the A_0.7_S_0.3_ coating was applied to a glass substrate and compared with a commercial white coating under sunlight. It indicates that the A_0.7_S_0.3_ coating achieved a surface temperature reduction of 3.0 °C compared to the commercial coating. On the right of Fig. 6(d), the A_0.7_S_0.3_ coating was also applied to a wooden board, with comparisons made between coated and uncoated substrates. It exhibited a 1.8 °C reduction in the coated wood compared to bare wood.

### 3.4 Durability of the A_0.7_S_0.3_ coatings

**Fig. 7:**
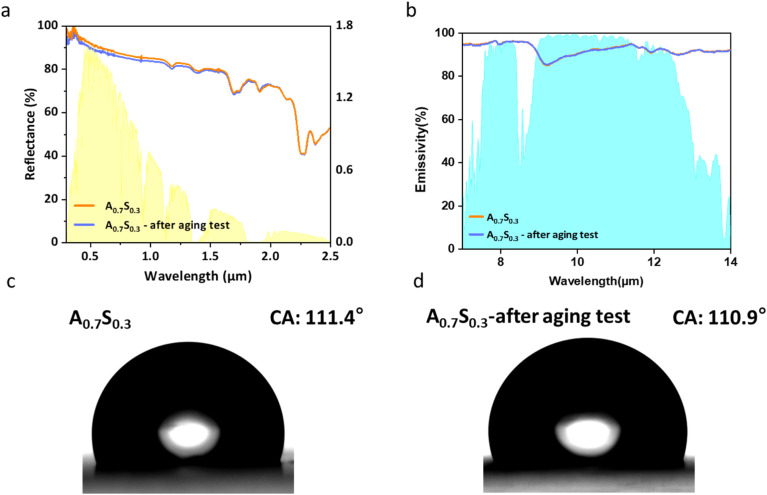
(a) Solar reflectance spectrum of A_0.7_S_0.3_ coatings before and after the aging test, (b) infrared emissivity spectrum of A_0.7_S_0.3_ coatings before and after the aging test, (c) water contact angle of original A_0.7_S_0.3_ coatings, and (d) water contact angle of A_0.7_S_0.3_ coatings after the aging test.

Radiative cooling coatings are highly susceptible to degradation caused by outdoor environmental factors such as UV exposure, elevated temperatures, and contamination from muddy water, all of which can limit their cooling performance.^64^ Therefore, the stability of radiative cooling coatings under outdoor conditions is critically important.^65,66^ To effectively evaluate the outdoor durability of the PMMA–PDMS coatings, we conducted an accelerated aging test similar to that in previous studies.^67,68^ The coatings were continuously exposed to UV light at a wavelength of 365 nm and an intensity of 8 mW cm^−2^ for 4 h, followed by thermal treatment at 80 °C for another 4 h. As shown in Fig. 7(a)–(c), the results of the outdoor aging test confirm that the PMMA–PDMS coatings can maintain optical performance and hydrophobicity comparable to the original coatings, even after exposure to intense UV irradiation and high-temperature treatment. This demonstrates the excellent durability of the coatings. Such stability is primarily attributed to the PMMA-1 particles, which provide high reflectance, particularly in the UV wavelength region, thereby effectively reducing solar absorption and mitigating UV-induced degradation.

## 4. Conclusion

In this study, we synthesized PMMA particles using two polymerization methods and confirmed their suitability for Mie scattering characteristics *via* comprehensive characterization. The PMMA-1 particles, with their optimized size for UV light scattering, demonstrated improved material properties, attaining an average reflectance of 93.7% and an emissivity of 93.2%. PMMA-1, combined with PDMS—a polymer with excellent radiative properties—was further developed into a fully polymer-based radiative cooling coating. Among the formulations, A_0.7_S_0.3_ exhibited the best performance, with an average solar reflectance of 96.9%, emissivity of 94.0%, and a water contact angle of 111.4°, indicating hydrophobicity. Moreover, outdoor experiments verified that, without reliance on reflective substrates like silver or aluminum, the A_0.7_S_0.3_ coating achieved an average temperature reduction of 3.4 °C below the ambient temperature under high solar irradiance (1005 W m^−2^) and humidity conditions (approximately 54.4%), with a maximum observed temperature difference of 8.6 °C. Infrared thermal imaging further showed a cooling advantage of 3.0 °C on glass substrates compared to commercial coatings, confirming the superior radiative cooling performance of this coating. Overall, we present a straightforward, cost-effective approach to developing high-performance A_0.7_S_0.3_ coatings that are applicable to a variety of substrates, maintaining effective cooling under sunlight. The hydrophobicity of the coating, demonstrated by its high-water contact angle, suggests self-cleaning potential, making this coating a promising candidate for practical applications in radiative cooling.

## Data availability

The data that support the findings of this study are available from the corresponding author upon reasonable request.

## Conflicts of interest

There are no conflicts to declare.

## Supplementary Material

RA-015-D5RA01834J-s001
