# Supplementary material for: Mie-optimized PMMA particles for fully polymer-based radiative cooling coatings with high reflectance and hydrophobicity
Source: RSC Adv. 2025 Jun 5;15(24):19104–15. doi: 10.1039/d5ra01834j (PMC12138783; doi:10.1039/d5ra01834j)
Supplement: RA-015-D5RA01834J-s001 [file RA-015-D5RA01834J-s001.pdf]

Supplementary Information (SI) for RSC Advances.
This journal is © The Royal Society of Chemistry 2025

Supplementary Materials

1. The comparison of different coatings

1.1. Different types of radiative cooling composite coatings

Table S1 Summary of optical properties and cooling performances of different types of radiative cooling composite coatings

Type	Filler	Binder	Reflectance	Emissivity	ΔT(°C)	Ref.
Hybrid	SiO_2/ Al_2O_3	Acrylic	94.1%	93.5%	7.9	2021 [1]
Hybrid	$BaSO_4$/ $CaCO_3$/ SiO_2	Acrylic	97.6%	N/A	8.3	2022 [2]
Hybrid	TiO_2@SiO_2	PDMS	95.0%	97%	5.3	2022 [3]
Hybrid	Raspberry-SiO_2	PDMS	N/A	N/A	9.7	2023 [4]
Hybrid	SiO_2	PVDF	95.0%	98.0%	10.5	2024 [5]
Hybrid	Al_2O_3	PMMA/TPU	97.7%	95.2%	4.9	2025 [6]
All polymer	PVDF	PDMS	90.6%	93.0%	4.9	2023 [7]
All polymer	PTFE	PDMS	92.0%	93.0%	4.7	2023 [8]
All polymer	PMMA	PDMS	96.9%	94.0%	3.4	This work

1.2. A comparison with PMMA-PDMS coating and commercial white coating

While both coatings target a thickness of 286-291 μm for consistent optical performance, the commercial paints typically require at least three spray applications to reach this thickness due to their limited film build-up per coat. In contrast, our formulation achieves the desired thickness in a single doctor blade process, reducing both material waste and labor. Moreover, the estimated material cost of the PMMA-PDMS coating is approximately $2.1/m^2$, which is lower than the $3.2/m^2$ required for commercial white coating (CWC), considering both raw reagent prices and process efficiency. These results highlight the practical advantages of our hybrid polymer coating system in terms of cost, application speed, and scalability for large-area radiative cooling surfaces.

Table S2 Comparison of coating methods, required thickness, slurry usage, and estimated cost between the PMMA-PDMS coating and commercial white coating

Sample	Method	Number of coats	Coating speed	Slurry volume	Thickness	Price
$A_{0.7}S_{0.3}$	Blade-coating	1	30 mm/s	1.25 cm^3	286 μm (5cm × 5 cm)	$2.1/m^2$
CWC	Spray coating	3	N/A	N/A	291μm (5cm × 5 cm)	$3.5/m^2$

2. Real Images

2.1. PMMA particle real images

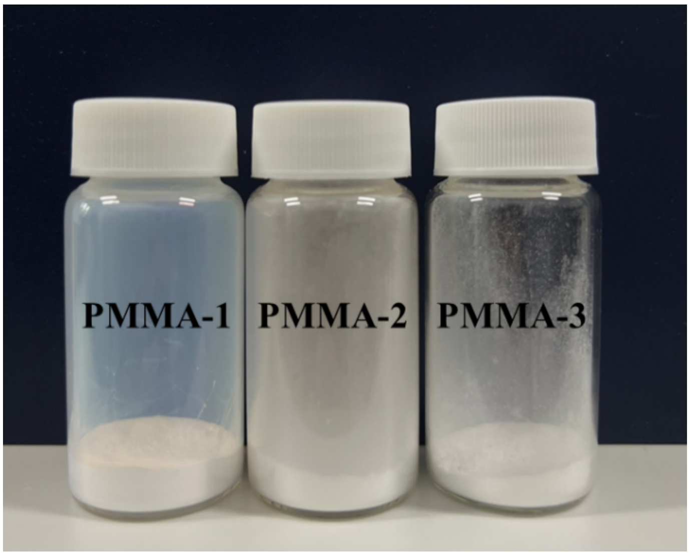

Fig. S1 PMMA-1 to PMMA-3 real images

2.2. PMMA-PDMS(A_XS_Y) coatings real images

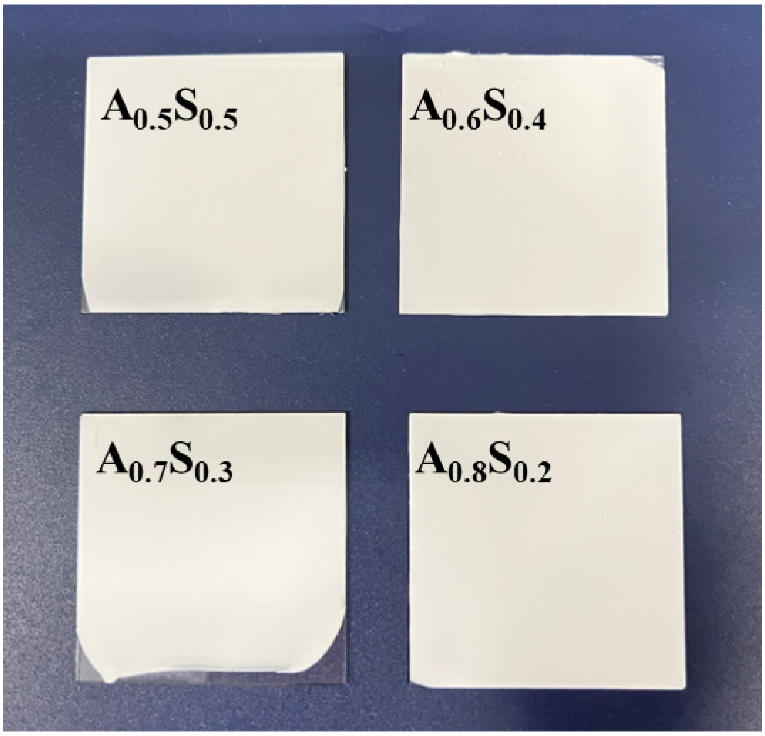

Fig. S2 $A_{0.5}S_{0.5}$ to $A_{0.8}S_{0}{\cdot}_{2}$ real images

2.3. Radiative cooling outdoor setup real images

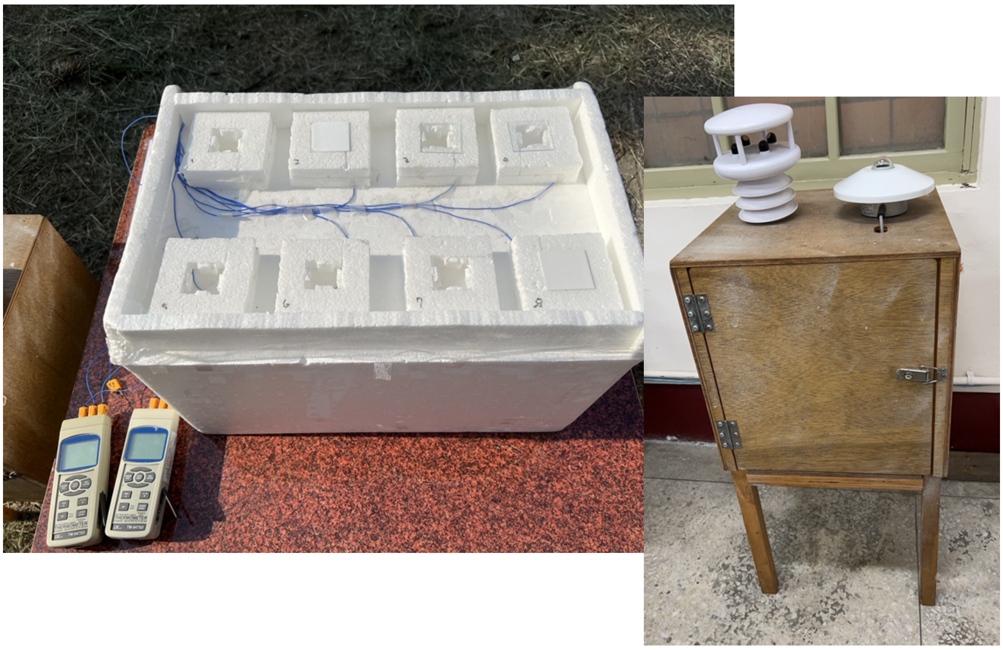

Fig. S3 Outdoor setup real images and instruments for climate measurement (solar irradiance, wind speed, and humidity) real images

3. Results and discussion

3.1. PMMA particle average reflectance and average emissivity

Table S3 PMMA-1 to PMMA-3 average reflectance and average emissivity

Sample	Reflectance(%)	Emissivity(%)
PMMA-1	93.7	93.2
PMMA-2	88.6	93.7
PMMA-3	87.4	93.6

3.2 $A_{0.5}S_{0.5}$ to $A_{0.8}S_{0.2}$ EDS-mapping top section

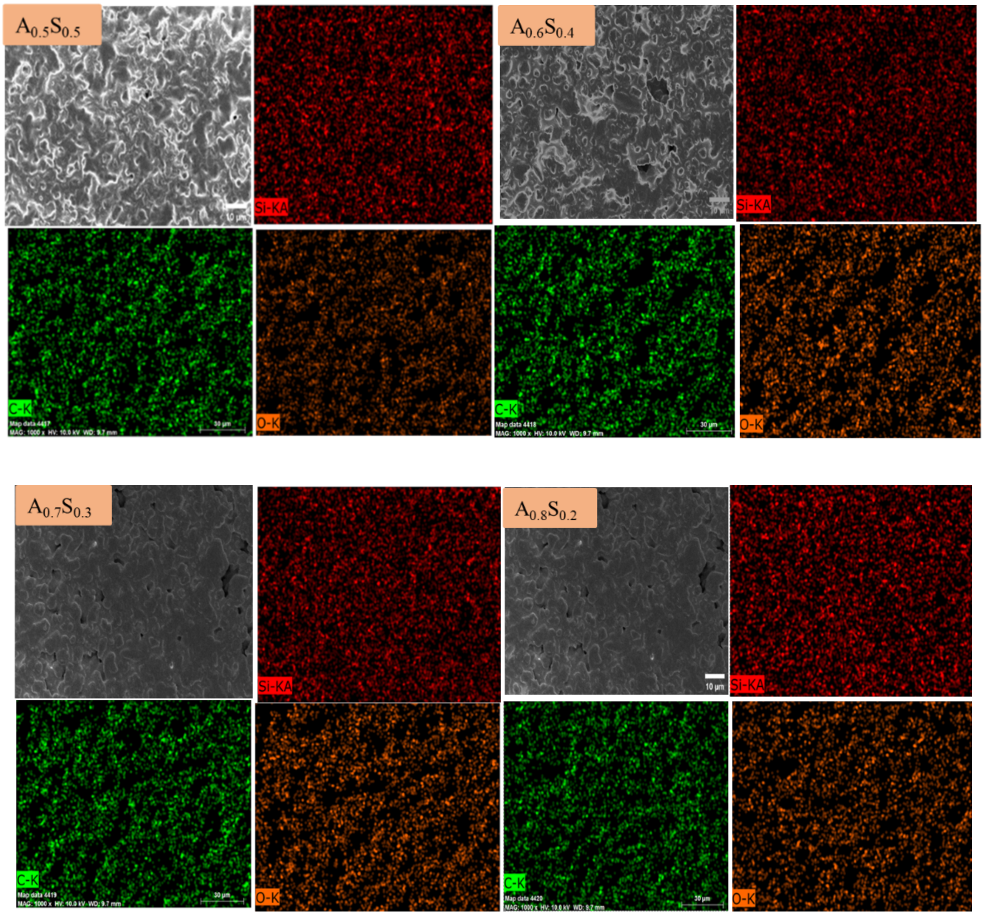

Fig. S4 $A_{0.5}S_{0.5}$ to $A_{0.8}S_{0.2}$ EDS-mapping top section

3.3 $A_{0.5}S_{0.5}$ to $A_{0.8}S_{0.2}$ EDS-mapping cross section

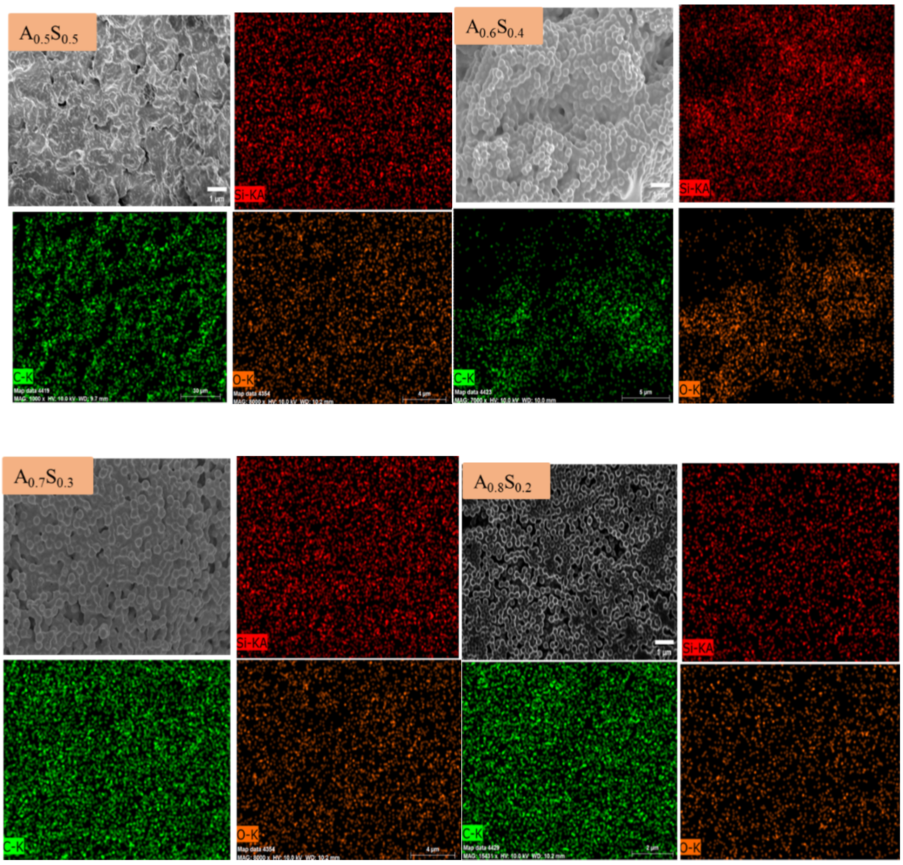

Fig. S5 $A_{0.5}S_{0.5}$ to $A_{0.8}S_{0.2}$ EDS-mapping cross section

3.4. $A_{0.5}S_{0.5}$ to $A_{0.8}S_{0.2}$ reflectance and average emissivity

Table S4 Average reflectance, average emissivity, and thickness of $A_{0.5}S_{0.5}$ to $A_{0.8}S_{0.2}$

Sample	Reflectance (%)	Emissivity (%)	Thickness (μm)
$A_{0.5}S_{0.5}$	79.0%	93.6%	286
$A_{0.6}S_{0.4}$	87.3%	95.4%	291
$A_{0.7}S_{0.3}$	96.9%	94.0%	286
$A_{0.8}S_{0.2}$	89.6%	93.7%	291
PMMA-1	93.7%	93.2%	N/A
PDMS	34.3%	94.4%	284
Commercial	79.7	94.9%	291

3.5. The PMMA-PDMS coatings were applied on various substrates

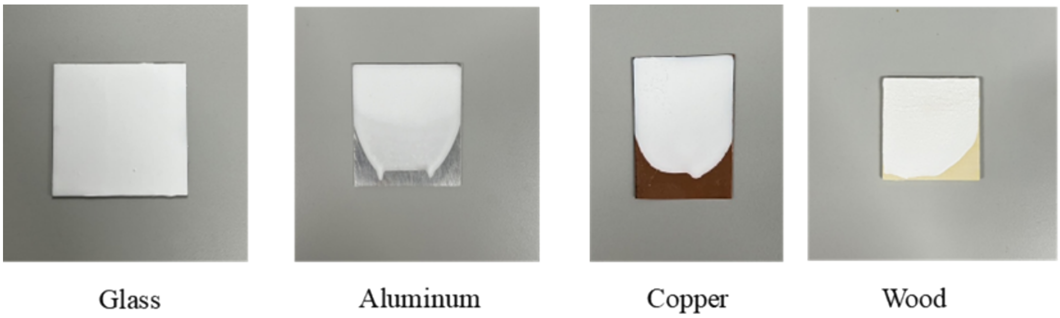

Fig. S6 The images of the PMMA-PDMS coatings on various substrates

References

(1) Chae, D.; Son, S.; Lim, H.; Jung, P. H.; Ha, J.; Lee, H. Scalable and paint-format microparticle–polymer composite enabling high-performance daytime radiative cooling. *Materials Today Physics* **2021**, *18*, 100389. DOI: https://doi.org/10.1016/j.mtphys.2021.100389.

(2) Dong, Y.; Han, H.; Wang, F.; Zhang, Y.; Cheng, Z.; Shi, X.; Yan, Y. A low-cost sustainable coating: Improving passive daytime radiative cooling performance using the spectral band complementarity method. *Renewable Energy* **2022**, *192*, 606-616. DOI: https://doi.org/10.1016/j.renene.2022.04.093.

(3) Hu, D.; Sun, S.; Du, P.; Lu, X.; Zhang, H.; Zhang, Z. Hollow Core-Shell Particle-Containing Coating for Passive Daytime Radiative Cooling. *Composites Part A: Applied Science and Manufacturing* **2022**, *158*, 106949. DOI: https://doi.org/10.1016/j.compositesa.2022.106949.

(4) Park, C.; Park, C.; Park, S.; Lee, J.; Kim, Y. S.; Yoo, Y. Hybrid emitters with raspberry-like hollow SiO2 spheres for passive daytime radiative cooling. *Chemical Engineering Journal* **2023**, *459*, 141652. DOI: https://doi.org/10.1016/j.cej.2023.141652.

(5) Wang, T.-Y.; Huang, C.-W. Harnessing optimized SiO_2 particles for enhanced passive daytime radiative cooling in thin composite coatings. *Solar Energy Materials and Solar Cells* **2024**, *278*, 113146. DOI: 10.1016/j.solmat.2024.113146.

(6) Park, J.; Chae, D.; Lim, H.; Ha, J.; Park, S.; Sung, H.; Park, C.; Lee, H. Daytime Radiative Cooling Sheet Functionalized by Al2O3-Assisted Organic Composite. *Advanced Science* **2025**, *12* (12), 2417584. DOI: https://doi.org/10.1002/advs.202417584 (acccessed 2025/05/16).

(7) Jiang, T.; Fan, W.; Wang, F. Long-lasting self-cleaning daytime radiative cooling paint for building. *Colloids and Surfaces A: Physicochemical and Engineering Aspects* **2023**, *666*, 131296. DOI: https://doi.org/10.1016/j.colsurfa.2023.131296.
(8) Jiang, T.; Lei, S.; Wang, F.; Ou, J.; Li, W.; Dai, R.; Dai, F.; Gu, Q.; Ni, W. All-Polymer Superhydrophobic Radiative Cooling Coating Based on Polytetrafluoroethylene/Polydimethylsiloxane Composites. *Industrial & Engineering Chemistry Research* **2023**, *62* (12), 5024-5034. DOI: 10.1021/acs.iecr.2c04448.
